# *pyActigraphy*: Open-source python package for actigraphy data visualization and analysis

**DOI:** 10.1371/journal.pcbi.1009514

**Published:** 2021-10-19

**Authors:** Grégory Hammad, Mathilde Reyt, Nikita Beliy, Marion Baillet, Michele Deantoni, Alexia Lesoinne, Vincenzo Muto, Christina Schmidt

**Affiliations:** 1 GIGA-CRC In vivo Imaging, University of Liège, Liège, Belgium; 2 Psychology and Neuroscience of Cognition, Faculty of Psychology, University of Liège, Liège, Belgium; Hebrew University of Jerusalem, ISRAEL

## Abstract

Over the past 40 years, actigraphy has been used to study rest-activity patterns in circadian rhythm and sleep research. Furthermore, considering its simplicity of use, there is a growing interest in the analysis of large population-based samples, using actigraphy. Here, we introduce *pyActigraphy*, a comprehensive toolbox for data visualization and analysis including multiple sleep detection algorithms and rest-activity rhythm variables. This open-source python package implements methods to read multiple data formats, quantify various properties of rest-activity rhythms, visualize sleep agendas, automatically detect rest periods and perform more advanced signal processing analyses. The development of this package aims to pave the way towards the establishment of a comprehensive open-source software suite, supported by a community of both developers and researchers, that would provide all the necessary tools for in-depth and large scale actigraphy data analyses.

## Author summary

The possibility to continuously record locomotor movements using accelerometers (actigraphy) has allowed field studies of sleep and rest-activity patterns. It has also enabled large-scale data collections, opening new avenues for research. However, each brand of actigraph devices encodes recordings in its own format and closed-source proprietary softwares are typically used to read and analyse actigraphy data. In order to provide an alternative to these softwares, we developed a comprehensive open-source toolbox for actigraphy data analysis, *pyActigraphy*. It allows researchers to read actigraphy data from 7 different file formats and gives access to a variety of rest-activity rhythm variables, automatic sleep detection algorithms and more advanced signal processing techniques. Besides, in order to empower researchers and clinicians with respect to their analyses, we created a series of interactive tutorials that illustrate how to implement the key steps of typical actigraphy data analyses. As an open-source project, all kind of user’s contributions to our toolbox are welcome. As increasing evidence points to the predicting value of rest-activity patterns derived from actigraphy for brain integrity, we believe that the development of the *pyActigraphy* package will not only benefit the sleep and chronobiology research, but also the neuroscientific community at large.

This is a *PLOS Computational Biology* Software paper.

## Introduction

Actigraphy consists in continuous movement recordings, using small watch-like accelerometers that are usually worn on the wrist or on the chest. As recordings can last several days or weeks, this technique is an adequate tool for in-situ assessments of the locomotor activity and the study of rhythmic rest-activity patterns. Consequently, it has been used in the field of sleep and circadian rhythm research [1] to assess night-to-night variability in estimated sleep parameters as well as rest-activity rhythm integrity. For example, intradaily variability has been associated with both cognitive and brain ageing [2, 3], while sleep fragmentation, as quantified by probability transitions from rest to activity during night-time, has been linked to cognitive performances [4] as well as to increased risks for Alzheimer’s disease [5].

However, the generalization of the findings made by this technique remains difficult; researchers either develop specific, often closed-source, data processing pipeline and/or analysis scripts, which are time-consuming, error prone and make the reproducibility of the analyses difficult, or they rely on commercial toolboxes that are not only costly but also act as black boxes. In addition, cumbersome manual data preprocessing, such as cleaning, hampers large scale analyses, which are mandatory for reliable and generalizable results.

Several initiatives to collect, host and share large actigraphy data sets have been successfully carried out over the past years; in 2012, the UK Biobank decided to add 7-day actimetry-derived physical activity data collection [6]. The National Sleep Research Resource [7] was launched in 2014 and it currently hosts actigraphy recordings for more than 18000 subjects. Not only these data sets were successfully used to perform genome-wide association studies, where the number of subjects is often a statistically limiting factor, and reveal links between rest-activity phenotypes and pathology of genetic background (e.g. [8, 9]) but they could also be crucial for understanding public health issues such as the impact of daylight time saving changes or chronic sleep deprivation. However, processing and analyzing such a large number of recordings remain a challenge. Therefore, the emergence of such biobanks should be matched by the emergence of appropriate analysis tools. Besides, facilitating the access to such analysis tools for actigraphy data would benefit other fields of neuroscience. For example, there are evidence for a link between human brain structure and the locomotor activity, whether it is the total amount of activity [10, 11], the sleep fragmentation [12] or the integrity of the circadian rhythmicity [3, 13]. Human brain functions are also modulated by circadian and/or seasonal rhythmicity [14, 15]. Therefore, a precise assessment of rhythmicity, as allowed by actigraphy, is crucial for functional brain imaging and cognitive studies too. These are only a few of the many examples that emphasize the benefit of extending the use of actigraphy outside the field of sleep and circadian research.

We thus argue that there is a need for a comprehensive and open-source toolbox for actigraphy data analysis. This motivated the development of the *pyActigraphy* package.

## Design and implementation

**Fig 1:**
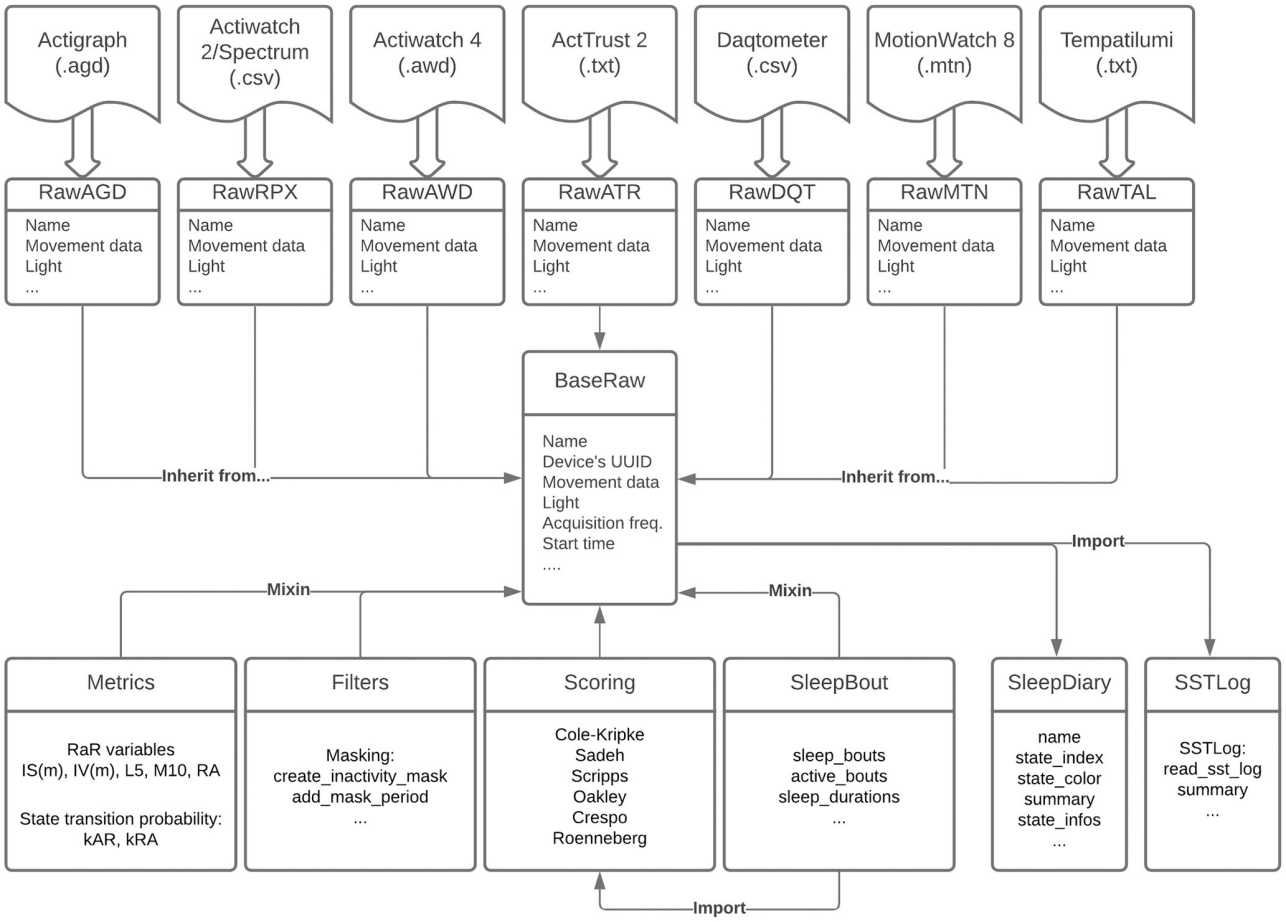
Diagram of the code architecture.

The *pyActigraphy* package is written in Python 3 (Python Software Foundation, https://www.python.org/). As illustrated in Fig 1, a dedicated class has been implemented for each file format to extract the corresponding actigraphy data, as well as the associated meta-data. These classes inherit from a base class implementing the various functionalities of the *pyActigraphy* package, via multiple inheritance (mixin). This centric approach provides multiple advantages; classes for new file formats can easily be implemented as they have to solely focus on reading the acquired data and meta-data. Additional functionalities will be inherited from the base class. In addition, newly added metrics or functions are readily available to all dedicated classes that derive from the base class. This design has been chosen in order to ease contributions from users with various coding skills.

Most of the variables and algorithms implemented in this package have been developed and validated for actigraphy devices that aggregate data into so-called movement counts. More recent devices provide now access to raw acceleration data and specific algorithms have been developed for this type of data (e.g [16, 17]). Nonetheless, it remains possible to convert these data into movement counts, providing a backward compatibility for these devices with algorithms validated with count-based devices [18]. This procedure has not yet been implemented in the *pyActigraphy* package but will be in the near future. However, data converted to counts can readily be used with our package.

### Reading native actigraphy files

The *pyActigraphy* package provides a unified way to read several actigraphy file formats. Currently, it supports output files from:

- wGT3X-BT, Actigraph (.agd file format only);
- Actiwatch 4 and MotionWatch 8, CamNtech;
- ActTrust 2, Condor Instruments;
- Daqtometer, Daqtix;
- Actiwatch 2 and Actiwatch Spectrum Plus, Philips Respironics;
- Tempatilumi, CE Brasil.

For each file format, a dedicated class has been implemented to extract the corresponding actigraphy data, as well as the associated meta-data. These classes inherit from a base class implementing the various functionalities of the *pyActigraphy* package. In addition, the package allows users to read actigraphy recordings, either individually, for visual inspection for example, or by batch, for analysis purposes.

### Masking and cleaning data

Before analysing the data, spurious periods of inactivity, where the actigraph was most likely removed by the participant, need to be discarded from the activity recordings. The *pyActigraphy* package implements a method to mask such periods, either manually or using timestamps specified in a text file. For convenience, it is also possible to automatically detect periods of continuous total inactivity, in order to create an initial mask that can be further visually inspected and edited by the users. Given that manual edition of masked periods might be tedious for large-scale data sets, more sophisticated methods for automatic masking [19, 20] could be implemented in the future. In addition to temporary actigraph removals, another usual source of artificial inactivities arises when the recordings start before and/or end after the actigraph is actually worn by the participant. Upon reading an actigraphy file, the *pyActigraphy* package allows users to discard such inactivity periods by specifying a start and a stop timestamp. The data collected outside this time range are not analyzed. These timestamps can also be specified by batch by using a simple log file where each line should correspond to the participant’s identification. This file is then processed to automatically apply such boundaries to the corresponding actigraphy file read by the package.

### Activity profile and onset/offset times

**Fig 2:**
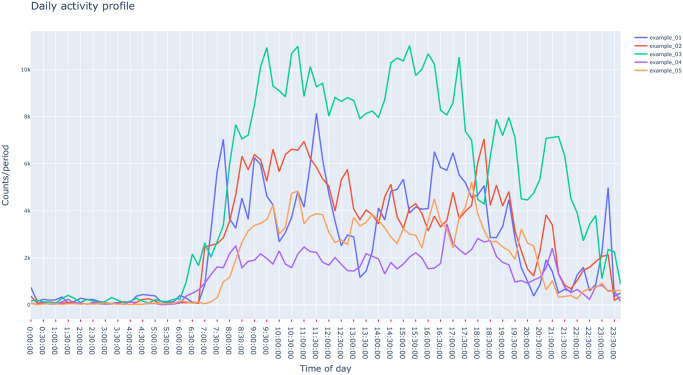
Visualization example of average daily profiles obtained with *pyActigraphy* using example files included in the package.

In circadian rhythm and sleep research, profile plots of the mean daily activity of actigraphy recording provides a visual tool to assess the overall rest-activity pattern, as well as recurrent behaviours such as naps. Patterns extracted from these profiles provide valid biomarkers that have been linked to cognitive decline [21] and psychiatric disorder [22]. Profiles are obtained by averaging consecutive data points that are 24h apart, over the consecutive days contained in the recording. The *pyActigraphy* package provides methods to construct these profiles (Fig 2). In addition, it provides methods to anchor the 24h-profile of an individual to a specific time and therefore ease group averaging; for example, if one uses the dim-light melatonin onset time, it becomes possible to compare activity data acquired at the same circadian phase across participants. For convenience, two methods have been implemented to detect the time points of a profile where the relative difference between the mean activity before and after this time point is maximal and minimal, respectively. These time points might then serve as initial estimates of the individual activity onset and offset times.

### Visualization of sleep agenda

**Fig 3:**
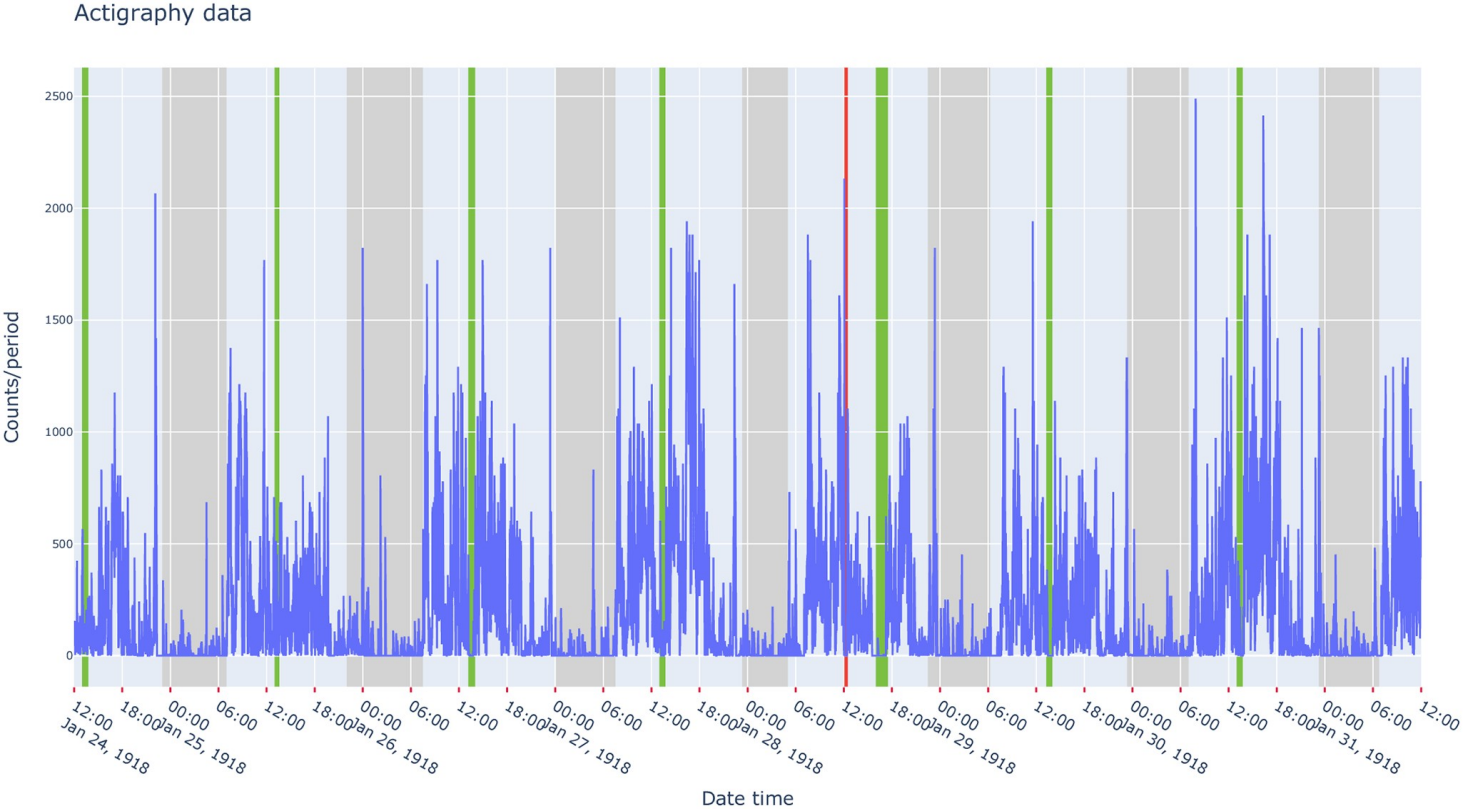
Visualization example of actigraphy data, overlaid with periods (green: Nap, grey: Night, red: Device not worn) reported in the sleep diary example file included in the package.

In both sleep research and medicine, a sleep diary is usually given with an actimeter to allow participants to report sleep episodes (duration and timing) as well as the subjective assessment of sleep quality for example. It allows comparisons between data recorded by an actigraph and the subjective perception of the individual wearing the device. In medical fields, sleep diaries are commonly recommended in order to help doctors in the diagnosis and treatment of sleep-wake disorders. The *pyActigraphy* package allows users to visualize and analyse sleep diaries, encoded as .ods or .csv files. Each row of these files indicates a new event, characterized by a type, a start time and an end time. A summary function provides descriptive statistics (mean, std, quantiles, …) for each type of events. For convenience and considering the current interests of the researchers involved in the development of the package, four types (active, nap, night, no-wear) are implemented by default when a sleep diary is read. However, the *pyActigraphy* package allows users to remove or customize these types and add new ones. As shown in Fig 3, the visualization of the sleep diary is allowed through the use of the python plotting library “plotly” [23]. Each event found in the sleep diary is associated with a plotly “shape” object that can be overlaid with the actigraphy data in order to visually assess the adequacy between the subjective reports and their objective counterparts.

### Rest-activity rhythm variables

Non-parametric rest-activity variables can easily be calculated with the *pyActigraphy* package. The list of such variables includes:

- the interdaily stability (IS) and the intradaily variability (IV) [24], which quantify the day-to-day variance and the activity fragmentation, respectively;
- the relative amplitude (RA) [25], which measures the relative difference between the mean activity during the 10 most active hours (M10) and the 5 least active ones (L5).

In addition, *pyActigraphy* implements the mean IS and IV variables, namely ISm and IVm [26], obtained by averaging IS or IV values calculated with data resampled at different frequencies. Finally, the *pyActigraphy* package allows users to calculate the values of the IS(m), IV(m) and RA variables for consecutive, non-overlapping time periods of user-defined lengths. Upon calling the corresponding function, users can specify the resampling frequency, if the data must be binarized before calculation, as well as the threshold used to binarize the data.

### Fragmentation of rest-activity patterns

The *pyActigraphy* package implements rest-activity state transition probabilities, *k*_*RA*_ and *k*_*AR*_ [27]. These variables quantify the fragmentation of the rest-activity pattern fragmentation; based on a probabilistic state transition model, where epochs with no activity are associated to a “rest” state (R) and to an “active” state (A) otherwise, the *k*_*RA*_ variable is associated with the probability to transition from a sustained “rest” state to an “active” state and the *k*_*AR*_ variable is associated with the probability to transition from a sustained “active” state to a “rest” state. The *pyActigraphy* package allows users to restrict the computation of the *k*_*RA*_ and *k*_*AR*_ variables to specific period of the day. For example, to target sleep periods, users may specify the activity offset and onset times (see section Activity profile and onset/offset times), as derived from individual activity profiles, as time boundaries. In the case of the *k*_*RA*_ variable, this would provide a quantification of the sleep fragmentation, adapted to a subject’s specific rest periods.

### Rest-activity period detection

The *pyActigraphy* package implements several rest-activity detection algorithms, which can be classified into two broad classes:

- Epoch-by-epoch rest/activity scoring algorithms: Cole-Kripke’s [28], Oakley’s [29], Sadeh’s [30] and Scripps’ [31] algorithms. The idea underlying these algorithms is to convolve the signal contained in a sliding window with a pre-defined kernel. Most algorithms use gaussian-like kernels. If the resulting value is higher than a certain threshold, then the epoch under consideration, usually the one located at the centre of the sliding window, is classified as active and as rest, otherwise. Finally, the window is shifted forward by one epoch and the classification procedure is repeated.
- Detection of consolidated periods of similar activity patterns: Crespo’s [32] and Roenneberg’s [33] algorithms. These two algorithms are fundamentally different from the epoch-by-epoch scoring algorithm as they intend to detect, at once, consolidated periods of rest. One advantage of this class of algorithms is that it provides a start and a stop time for each period classified as rest.

**Fig 4:**
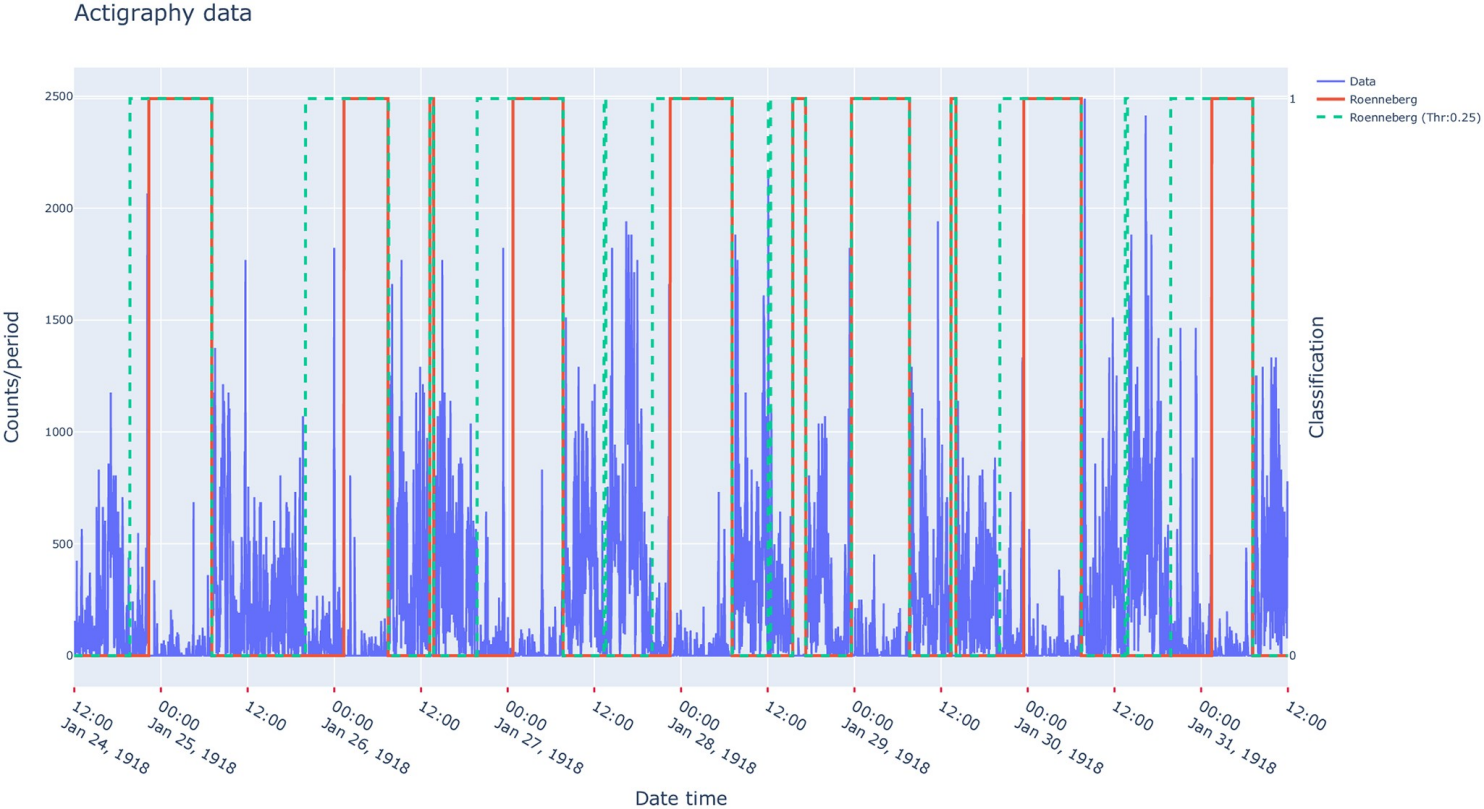
Visualization example of actigraphy data, overlaid with periods scored as “active” (0) or “rest” (1) by Roenneberg’s algorithm [33] for two different settings (full line: Default parameter values, dash line: With a threshold set at 0.25 of the activity trend).

As illustrated in Fig 4, these algorithms have been implemented to return a binary time series: 0 being rest or activity depending on the definition made in the original article describing the detection algorithm.

Based on the aforementioned algorithms, the *pyActigraphy* package allows also the computation of a sleep regularity profile which quantifies the probability for the participant to be in the same state (rest or active) at any daytime point on a day-by-day basis. From this 24h profile, the sleep regularity index (SRI) [34, 35] can be calculated as the product of theses probabilities over all the time bins. Finally, using the detection algorithms of the latter class, the *pyActigraphy* package allows the computation of the sleep midpoint as described in [35].

### Advanced signal processing

The *pyActigraphy* package makes available additional functions for more advanced analyses of actigraphy recordings:

- Cosinor [36]: the idea of a Cosinor analysis is to estimate some key parameters of the actigraphy count series by fitting these data with a (co)sine curve:
  $$Y\left(t\right)=M+A*cos\left(\frac{2\pi }{T}*t+\phi \right)$$
  where M is the MESOR (Midline Statistic Of Rhythm), A is the amplitude of the oscillations, T is the period and *ϕ* is the acrophase. The fit procedure provides estimates of these parameters which can then help to characterize the 24h rest-activity rhythm of an individual.
- Detrented Fluctuation Analysis (DFA) [37, 38]: human activity exhibits a temporal organization characterised by scale-invariant (fractal) patterns over time scales ranging from minutes to 24 hours. This organization has been shown to be degraded with aging and dementia [39]. The DFA method allows the quantification of this scale-invariance and comprises four steps:
  1. Signal integration and mean subtraction
  2. Signal segmentation
  3. Local detrending of each segment
  4. Computation of the q-th order fluctuations
  All these steps have been implemented in the DFA class of *pyActigraphy*.
- Functional linear modelling (FLM) [40]: it consists in converting discrete measures to a function or a set of functions that can be used for further analysis. In most cases, the smoothness of the resulting function is under control, which ensures the derivability of this function. Three techniques are available in *pyActigraphy* to convert the actigraphy data to a functional form:
  - Fourier expansion
  - B-spline interpolation
  - Smoothing
  In the context of actigraphy, functional linear modelling and analysis have been successfully applied to link sleep apnea and obesity to specific circadian activity patterns [41].
- Locomotor inactivity during sleep (LIDS) [42]: the analysis of the locomotor activity during sleep revealed a rhythmicity that mimics the ultradian dynamic of sleep. This type of analysis opens new opportunities to study, *in situ*, sleep dynamics at a large scale and over large individual time periods. The LIDS class implements all the necessary functions to perform the analysis of the LIDS oscillations:
  - sleep bout filtering
  - non-linear conversion of activity to inactivity
  - extraction of the characteristic features of the LIDS oscillations via a cosine fit
- Singular spectrum analysis (SSA) [43, 44]: this technique allows the decomposition of a time series into additive components and the quantification of their respective partial variance. In the context of actigraphy, SSA can be used to extract the signal trend as well as circadian and ultradian components separately. The latter is relevant in human sleep research because sleep is not only alternating with wakefulness over the 24-hour cycle, but also exhibits an ultradian modulation, as mentioned previously. For example, a SSA analysis has been used to reveal alterations of the ultradian rhythms in insomnia [45]. All the necessary steps for the SSA and related functions, namely the embedding, the singular value decomposition, the eigentriple grouping and the diagonal averaging, are implemented in the SSA class. Since the subsequent calculations can be computationally intensive, the class implementation uses the open-source compiler Numba [46] for a direct translation of the functions to machine code and therefore improve their execution speed by several orders of magnitudes.

### Online documentation and tutorials

The online documentation of the *pyActigraphy* package (https://ghammad.github.io/pyActigraphy) contains instructions to install the package, as well as information about the authors and the code license. It also contains a list of the attributes and methods available in the *pyActigraphy* package. More information about their implementation, as well as the reference to the related original research articles, can be found in the online API documentation (https://ghammad.github.io/pyActigraphy/api.html), which is generated automatically from source code annotations. In order to keep the documentation up to date with the latest developments of the package, the documentation is automatically generated anew and made available online for each new release. Finally, the online documentation offers several tutorials (https://ghammad.github.io/pyActigraphy/tutorials.html), illustrating the various functionalities of the package. These tutorials are generated from Jupyter notebooks [47] that are included in the *pyActigraphy* package itself, so that they can be used by any user to reproduce and practice the various functionalities of the *pyActigraphy* package in an interactive and user-friendly environment. As input data, the tutorials use real example data files that are included in the package for illustration and testing purposes. In total, 13 examples are included.

### Continuous integration and automated unit tests

The development of the *pyActigraphy* follows practices intended to reduce the probability of coding errors such as continuous integration [48]. For the integration of a new feature in the package, a suite of unit tests is run with the *pytest* (https://docs.pytest.org/en/latest) framework and, upon success only, this feature is permanently integrated to the package. There are currently more than 50 different tests in the test suite, covering various functionalities of the *pyActigraphy* package. For example, for each supported file format, an example file, with known information in its header, is available in the package and the associated unit tests ensure that the corresponding reader function is able to retrieve the correct information from that file. In addition, synthetic data (ex: sine and square waves with known periods, as well as Gaussian noise) are used to test the implementations of variables such as IS, IV, *k*_*RA*_, *k*_*AR*_ for which the value can be analytically derived. For example, the IS is equal to 1 and IV equal to 0 for any periodic data with a period of 24h. In case of data whose series of consecutive zeros follow a geometric distribution with a probability of success *p*, the *k*_*RA*_ is equal to *p*. Finally, to assert further the correct implementation of the rest-activity rhythm variables, values obtained with the *pyActigraphy* package have been compared with values obtained with a commercial software (MotionWare, version 1.2.28), using the file “example01.AWD” included in the *pyActigraphy* package. The analysis uses 7 days of data, starting from the 25/01/1918 at 00:00 AM. As shown in Table 1, differences, if any, are below 0.2% and are thus most likely due to round-off errors.

**Table 1:** Values of the rest-activity rhythm variables obtained with Motionware (1.2.28) and *pyActigraphy*.

Variable	MotionWare (1.2.28)	*pyActigraphy*
IS	0.622	0.623
IV	0.788	0.788
RA	0.918	0.919
L5	13.37^1^	13.37
L5 (start time)	02:00 AM	02:00 AM
M10	311.97^1^	311.97
M10 (start time)	08:00 AM	08:00 AM

^1^Mean hourly values obtained with MotionWare have been divided by 60 to match the definition used in *pyActigraphy* (mean value per acquisition epoch)

## Results

Most actigraphy devices encode their data in proprietary format. Therefore, these devices are bound to their specialized commercial software to read and analyse the acquired data. While softwares like MotionWare (CamNtech, Cambridge, UK), ActStudio (Condor Instruments, São Paolo, Brasil), Actilife (ActiGraph LLC, Pensacola, FL) or Actiware (PhilipsRespironics, Murrysville, PA) give access to a variety of activity and sleep parameters, such as total sleep time, sleep onset, etc, they provide very limited views on their current implementations of the algorithms applied to the data and very little possibilities to apply new ones. Over the last years, there have been efforts to create open-source analysis tools; from packages to simply calculate the IS, IV and RA variables (nparACT [49]) to softwares allowing the preprocessing of large accelerometer data sets and the extraction of the sleep timing and duration (e.g. biobankAccelerometerAnalysis [6, 50–52], GGIR [53], OMGUI [54]). However, to our knowledge, there is no comprehensive open-source analysis package for actigraphy data that would allow users to read various data format, perform the necessary data cleaning as well as more advanced data analysis within a single framework in the python ecosystem. This is all the more necessary as it would improve the reproducibility of research outcomes by limiting the proliferation of private analysis codes [55]. It would also allow users to perform more complex analyses and therefore make optimal use of actigraphy data that are often part of costly multi-modal data acquisition protocols. Such analysis package would also help to reduce error rates by alleviating the burden of manual data processing that hampers the processing of large-scale actigraphy data sets. So far, the *pyActigraphy* package has successfully been used to compute non-parametric rest-activity rhythm variables [56, 57], to automatically detect sleep periods with multiple algorithms [58, 59] or assess sleep fragmentation via transition state probability [60].

In order to facilitate the understanding of the various functionalities of the package and to help researchers to design their analysis code, tutorial notebooks are available. They are divided into three categories, with an increasing complexity;

1. Introductory notebooks:
  - Intro: this notebook introduces how to read an actigraphy file and retrieve some of the most common meta-data that are useful for subsequent analyses. It also shows how to plot the raw actigraphy data for visual inspection.
    Link to the corresponding tutorial: https://ghammad.github.io/pyActigraphy/pyActigraphy-Intro.html
  - Batch: this notebook illustrates how to read files by batch and how to access information (meta-data or metrics) of all these files at once.
    Link to the corresponding tutorial: https://ghammad.github.io/pyActigraphy/pyActigraphy-Batch.html
2. Feature notebooks:
  - Masking: this notebook provides informations about how to automatically mask spurious inactivity periods, during which the device has most likely been removed by the participant. The impact of such masking on the calculation of rest-activity variables is also shown.
    Links to the corresponding tutorials: https://ghammad.github.io/pyActigraphy/pyActigraphy-Masking.html
  - SSTLog: this notebook highlights the possibility with *pyActigraphy* to remove, in an automatic fashion, the start and end periods of recordings read by batch. The need to remove such periods arise quite often when the device is set to acquire data while it is not yet worn or not worn anymore, by the participant.
    Links to the corresponding tutorials: https://ghammad.github.io/pyActigraphy/pyActigraphy-SSt-log.html
  - RaR: this notebook demonstrates how to calculate, for an individual file or by batch of files, various rest-activity rhythm variables, such as IS, IV, ISm, IVm, etc. Effects of resampling, binarization or thresholding on these variables are also shown.
    Link to the corresponding tutorial: https://ghammad.github.io/pyActigraphy/pyActigraphy-Non-parametric-variables.html
  - SleepDiary: this notebook shows how to overlay the actigraphy data with the different periods reported in its corresponding sleep diary. It uses a sleep diary example provided with the package to illustrate the accepted diary format and how to custom its layout.
    Link to the corresponding tutorial: https://ghammad.github.io/pyActigraphy/pyActigraphy-Sleep-Diary.html
  - RestAlgo: in this notebook, the six different rest period detection algorithms implemented in *pyActigraphy* are reviewed; a distinction is made between algorithms providing an epoch-by-epoch rest-activity scoring and algorithms aiming to detect consolidated periods of rest or activity.
    Link to the corresponding tutorial: https://ghammad.github.io/pyActigraphy/pyActigraphy-Sleep-Algorithms.html
  - SleepFrag: this notebook is an illustration of the state transition probability model implemented in *pyActigraphy*; this model is used to quantify the probability to transition from a “rest” state to an “active” state. By restricting the time window of the data used to estimate this probability to the habitual night time, this notebook illustrates how to derive an index of sleep fragmentation.
    Link to the corresponding tutorial: https://ghammad.github.io/pyActigraphy/pyActigraphy-StateTransitionProb.html
3. Analysis notebooks:
  - Cosinor: in this notebook, instructions are given to perform a Cosinor analysis and retrieve the associated fit parameters, namely the mesor, the period, the acrophase, etc. Analyses of a single actigraphy file and of a batch of files are both illustrated. This notebook also contains words of caution about the use of a Cosinor analysis with non-stationary actigraphy data.
    Link to the corresponding tutorial: https://ghammad.github.io/pyActigraphy/pyActigraphy-Cosinor.html
  - FLM: this notebook provides examples about how to obtain a smooth representation of the inherently noisy actigraphy data, either with a basis function expansion technique (Fourier functions or B-splines) or with a Gaussian kernel function. Examples for group analysis of multiple files at once are also provided.
    Link to the corresponding tutorial: https://ghammad.github.io/pyActigraphy/pyActigraphy-FLM.html
  - MF-DFA: this notebook illustrates the functions implemented in *pyActigraphy* to perform the different steps of a (multi-fractal) detrended fluctuation analysis; from signal integration to estimation of the generalized Hurst exponent.
    Link to the corresponding tutorial: https://ghammad.github.io/pyActigraphy/pyActigraphy-MFDFA.html
  - SSA: in this last notebook, the SSA methodology is reviewed. As an illustration, the signal from an example file is decomposed into a trend, a circadian component and an ultradian component. This notebook also provides indications about how to use a weighted-correlation matrix to group the elementary matrices, prior to reconstructing the different components of the signal.
    Link to the corresponding tutorial: https://ghammad.github.io/pyActigraphy/pyActigraphy-SSA.html

## Availability and future directions

The *pyActigraphy* package has been released under the GPLv3 license and is available from the Python Package Index (PyPI) repository: https://pypi.org/project/pyActigraphy. Its source code is hosted by Github (https://github.com/ghammad/pyActigraphy) and the Zenodo platform [61]. The online documentation of the *pyActigraphy* package contains a detailed description of the attributes and methods of its various modules and is meant to be used as complementary material of the current paper. In addition, more than a dozen of tutorials are made available online to illustrate how to use the multiple features of the package, described in this paper. By developing the *pyActigraphy* package, we not only hope to facilitate data analysis but also foster research using actimetry and drive a community effort to improve this open-source package and develop new variables and algorithms. As such, user’s contributions of any type (code, documentation, suggestion of new features, etc.) are welcome and encouraged.
